# Circulating Tumor DNA Genomic Profiling in ^223^Ra-Treated Metastatic Castration-Resistant Prostate Cancer: The KYUCOG-1901 Study

**DOI:** 10.2967/jnumed.126.272073

**Published:** 2026-07

**Authors:** Masaki Shiota, Maki Fujiwara, Takayuki Sumiyoshi, Hideki Enokida, Tomomi Kamba, Tsukasa Igawa, Naoya Masumori, Hirotsugu Uemura, Toshiyuki Kamoto, Katsuyoshi Higashijima, Kensuke Mitsunari, Hiroji Uemura, Takashi Kobayashi, Shusuke Akamatsu, Shoji Tokunaga, Takuro Isoda, Kousei Ishigami, Masatoshi Eto

**Affiliations:** 1 Department of Urology, Graduate School of Medical Sciences, Kyushu University, Fukuoka, Japan;; 2 Department of Urology, Graduate School of Medicine, Kyoto University, Kyoto, Japan;; 3 Department of Urology, Graduate School of Medical and Dental Sciences, Kagoshima University, Kagoshima, Japan;; 4 Department of Urology, Graduate School of Medical Sciences, Kumamoto University, Kumamoto, Japan;; 5 Department of Urology, School of Medicine, Kurume University, Kurume, Japan;; 6 Department of Urology, School of Medicine, Sapporo Medical University, Sapporo, Japan;; 7 Department of Urology, Faculty of Medicine, Kindai University, Osaka-Sayama, Japan;; 8 Department of Urology, Faculty of Medicine, University of Miyazaki, Miyazaki, Japan;; 9 Department of Urology, School of Medicine, University of Occupational and Environmental Health, Kitakyushu, Japan;; 10 Department of Urology, Graduate School of Biomedical Sciences, Nagasaki University, Nagasaki, Japan;; 11 Department of Urology and Renal Transplantation, Yokohama City University Medical Center, Yokohama, Japan;; 12 Department of Urology, Graduate School of Medicine, Nagoya University, Nagoya, Japan;; 13 Clinical Therapeutic Trial Center, Ehime University Hospital, Japan; and; 14 Department of Clinical Radiology, Graduate School of Medical Sciences, Kyushu University, Fukuoka, Japan

**Keywords:** circulating tumor DNA, ctDNA fraction, genomic profile, liquid biopsy, ^223^Ra

## Abstract

Circulating tumor DNA (ctDNA) testing has emerged as a cancer precision medicine approach. We investigated the genomic landscape and clinical utility of ctDNA in patients receiving ^223^Ra dichloride for bone metastatic castration-resistant prostate cancer (mCRPC). **Methods:** This prospective, observational, multicenter study enrolled patients treated with ^223^Ra for bone mCRPC. Targeted sequencing of cell-free DNA from plasma at baseline and end of treatment (EOT), along with paired leukocyte DNA, was performed using an 88-gene panel. Associations between ctDNA profiles and clinical outcomes, including biomarker response, radiographic progression-free survival (rPFS), and overall survival (OS), were analyzed. **Results:** Of 93 patients analyzed, ctDNA was successfully profiled in 84 baseline and 74 EOT samples, with matched data available for 68 patients. A ctDNA fraction of at least 5%, as well as *TP53* alteration, *PTEN* alteration, and cell cycle pathway alterations at baseline were significantly associated with shorter rPFS and OS. Dynamic changes in ctDNA fraction and *PTEN* alteration between baseline and EOT correlated with distinct rPFS and OS. **Conclusion:** This study suggests the clinical utility of ctDNA profiling as both a prognostic and a monitoring tool in patients with bone mCRPC treated with ^223^Ra. The findings obtained in this study raise the possibility that ctDNA could contribute to future strategies for risk stratification or treatment monitoring during ^223^Ra therapy.

## Visual Abstract

**Figure:**
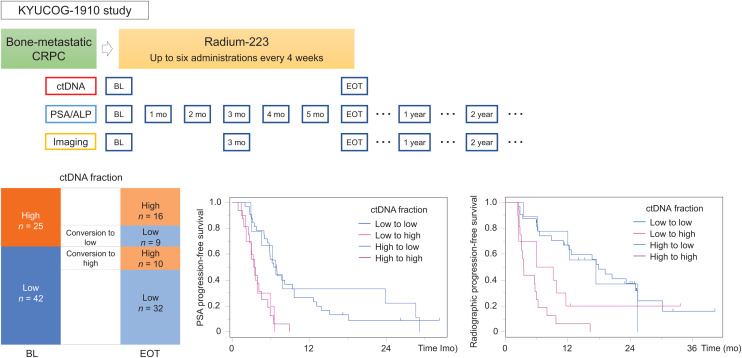

Prostate cancer is one of the most commonly diagnosed malignancies in men in Western countries (1). Metastatic castration-sensitive prostate cancer arises either de novo or as a recurrence after radical local therapy for localized disease, accounting for approximately 10% of newly diagnosed prostate cancer cases in Japan (2). Although first-line androgen deprivation therapy is initially highly effective, alleviating cancer-related symptoms, lowering prostate-specific antigen (PSA) levels, and reducing tumor burden, therapeutic resistance inevitably develops, and most metastatic castration-sensitive prostate cancer cases eventually progress to metastatic castration-resistant prostate cancer (mCRPC). ^223^Ra dichloride is a bone-targeted radionuclide therapy shown to improve overall survival (OS) in patients with bone mCRPC (3). In addition, ^223^Ra reduces skeletal-related events and improves quality of life, as supported by real-world evidence (4,5). However, clinical outcomes with ^223^Ra vary among patients, and no reliable biomarker has been established to predict or monitor treatment response. Thus, there remains an unmet need for robust prognostic and monitoring biomarkers in patients receiving ^223^Ra.

Circulating tumor DNA (ctDNA) testing has emerged as a promising approach to advance precision oncology. Although next-generation sequencing of tumor tissue remains the gold standard for comprehensive genomic profiling, obtaining biopsy samples from metastatic sites is often challenging. Moreover, a single-site tissue biopsy may not adequately capture intrapatient tumor heterogeneity. In contrast, ctDNA can be collected less invasively and repeatedly, providing a real-time genomic snapshot of the tumor and its heterogeneity (6–8). Increasing evidence supports the clinical utility of ctDNA analysis, demonstrating its predictive value for treatment outcomes and its ability to track clonal evolution under therapeutic pressure in patients receiving androgen receptor signaling inhibitors or taxanes (9–11). However, data on ctDNA profiling in the context of ^223^Ra therapy remain scarce. Therefore, we conducted a prospective, observational study to investigate the genomic landscape and clinical utility of ctDNA in patients with bone mCRPC treated with ^223^Ra.

## MATERIALS AND METHODS

### Study Design

The KYUCOG-1901 study was a prospective, observational, multicenter study conducted at 19 institutions across Japan (12). It aimed to investigate ctDNA genomic profiling and single nucleotide polymorphisms (SNPs) in patients receiving ^223^Ra for bone mCRPC.

Key inclusion criteria were histopathologically confirmed prostatic adenocarcinoma, evidence of castration-resistant progression while on castration or continuous androgen deprivation therapy, at least 2 bone metastatic lesions, Eastern Cooperative Oncology Group performance status 0–2, adequate organ function, and expected survival of at least 6 mo. Key exclusion criteria included prior radionuclide therapy (e.g., ^89^Sr) within 6 mo, prior ^223^Ra, active malignancy other than prostate cancer, presence or history of visceral or brain metastases, lymph node metastases of at least 1.5 cm (short axis), and spinal cord compression.

The study was conducted in accordance with the Declaration of Helsinki and the Japanese Ethical Guidelines for Medical and Health Research Involving Human Subjects. Written informed consent was obtained from all patients. The protocol was approved by the Certified Review Board and registered in the University Hospital Medical Information Network Clinical Trials Registry (UMIN000040358, posted on May 11, 2020). The target sample size was 100 patients, with a planned follow-up of 2 y from treatment initiation. The study began in May 2020, enrollment completed in April 2023, and data were cut off on April 30, 2025.

Patients received up to 6 intravenous injections of ^223^Ra (55 kBq/kg) every 4 wk. Concomitant and subsequent treatments were at the discretion of the treating physicians. The primary endpoint was the change in ctDNA levels and frequency of genomic alterations during ^223^Ra therapy. Secondary endpoints included associations between ctDNA features and changes in PSA and alkaline phosphatase (ALP) levels, time to PSA and ALP progression, and radiographic progression-free survival (rPFS) and OS.

Changes in PSA and ALP levels were defined as the maximum percentage decrease or minimum percentage increase relative to baseline during treatment. PSA–progression-free survival (PFS) was defined as the time from enrollment to progression, determined by an increase of at least 25% and 2 ng/mL from nadir (confirmed ≥3 wk later) or by an increase of at least 25% and 2 ng/mL after 12 wk if no initial decline occurred. The bone scan index (BSI) at baseline and 4 wk after last treatment was calculated using commercially available software at each institution. Neural network–based algorithms automatically segmented anatomic regions of the skeleton, detected and classified abnormal hot spots, and calculated the weight fraction of skeletal involvement (13). ALP-PFS was defined as the time from enrollment to progression, determined by an increase of at least 25% from nadir (confirmed ≥3 wk later) or by an increase of at least 25% after 12 wk if no initial decline occurred. rPFS was defined as the time from enrollment to radiographic progression (per RECIST version 1.1 and Prostate Cancer Clinical Trials Working group 3 criteria) or death. OS was defined as the time from enrollment to death from any cause (14,15).

### ctDNA Analysis

Approximately 10 mL of blood were collected within 4 wk before the first treatment and within 4 wk after the final administration for ctDNA analysis using PAXgene circulating cell-free DNA (cfDNA) tubes (PreAnalytiX). ctDNA genotyping was performed as previously described (9). A median of 12 ng of cfDNA (interquartile range [IQR], 10–12 ng) and 50 or 100 ng of matched leukocyte DNA were used for library preparation with ThruPLEX Tag-seq (Takara Bio). Targeted sequencing was performed with the KAPA HyperChoice system (Roche) on an Illumina NovaSeq platform, covering exonic regions of 88 genes (Supplemental Table 1 [supplemental materials are available at http://jnm.snmjournals.org]). Sequencing data were processed using the eVIDENCE pipeline for somatic mutation identification (9). Somatic mutations were called in cfDNA by searching for variants with at least 5 unique sequencing reads and a variant allele fraction (VAF) of at least 0.5%. To eliminate artifacts and germline SNPs, reads detected in paired leukocyte DNA with a VAF of at least 1% were discarded. For variants with a leukocyte VAF of less than 1%, a 1-sided Fisher exact test was applied; variants with a significantly higher VAF in cfDNA (*P* < 0.001) were considered tumor-derived. Recurrent artifacts (>3 samples) were excluded unless they were known prostate cancer hot spots (according to the COSMIC database; Wellcome Sanger Institute) or pathogenic or likely pathogenic (according to the ClinVar archive; National Center for Biotechnology Information) (16,17). The protein-level consequences of the mutations were predicted using ANNOVAR (University of Southern California) (18). Loss-of-function (nonsense, frameshift, or splice-site) variants and pathogenic or likely pathogenic missense variants (according to COSMIC or ClinVar) were considered deleterious. In addition, missense variants identified in COSMIC as recurrent variants in mCRPC or classified as pathogenic or likely pathogenic in ClinVar were classified as deleterious (16,17).

Copy number alterations were analyzed from cfDNA and leukocyte DNA with CNVkit (version 0.9.6; University of California, San Francisco) (19). Pooled reference data were created by assessing all leukocyte samples, and coverage log ratios were calculated against the reference data. For gene copy number calling, we visualized the association between the median allele fraction of heterozygous SNPs and the coverage log ratio of all target genes, referring to several reports (Supplemental Fig. 1) (20,21). Copy number deletions and gains of target genes, except for the following 6 genes, were defined by log ratios of no more than −0.5 and of at least 0.25, respectively; deep deletions and amplifications were defined by log ratios of no more than −1.0 and of at least 1.0, respectively. For *CDKN1B*, the thresholds for shallow and deep deletion were changed to −1.0 and −1.5, respectively. For *BRCA2, MSH6, ZFHX3,* and *APC*, the thresholds for shallow and deep deletion were changed to −0.6 and −1.1, respectively. For *MYC*, the thresholds for shallow deletion, gain, and amplification were changed to −0.6, 0, and 0.6, respectively.

We applied 2 orthogonal methods to estimate the ctDNA fraction: leverage of the VAF of somatic mutations and deviation in the b-allele fraction of heterozygous germline SNPs (9,22). For cfDNA samples harboring at least 1 somatic mutation, the ctDNA fraction was estimated using the highest somatic VAF per autosomal variant or VAF for chromosome X variants, with adjustment to exclude amplified genes. For samples without detectable variants but with copy number alterations, the ctDNA fraction was alternatively estimated from heterozygous SNP allele frequencies in monoallelic deleted regions.

### Statistical Analyses

Statistical analyses were conducted using JMP 17 (SAS Institute). Continuous and categoric variables were summarized as median with IQR in parentheses and number with percentage in parentheses, respectively. Between-group comparisons used the Wilcoxon rank-sum test for continuous data and Fisher exact test for categoric data. Thresholds for the cfDNA amount and ctDNA fraction were determined by receiver operating characteristic curve analysis, using any PSA decline as the reference outcome. A ctDNA fraction cutoff of 5% was selected, because it provided the optimal balance of sensitivity and specificity. The association between baseline ctDNA fraction and BSI was evaluated using the Spearman rank correlation coefficient. Survival curves were estimated by the Kaplan–Meier method and compared by a log-rank test. Cox proportional hazards models were used for univariate and multivariate analyses to calculate hazard ratios (HRs) with 95% CIs. Paired categoric data were analyzed using the McNemar test. All *P* values were 2-sided, and a *P* value of less than 0.05 was considered statistically significant.

## RESULTS

### Patient Backgrounds

**FIGURE 1.**
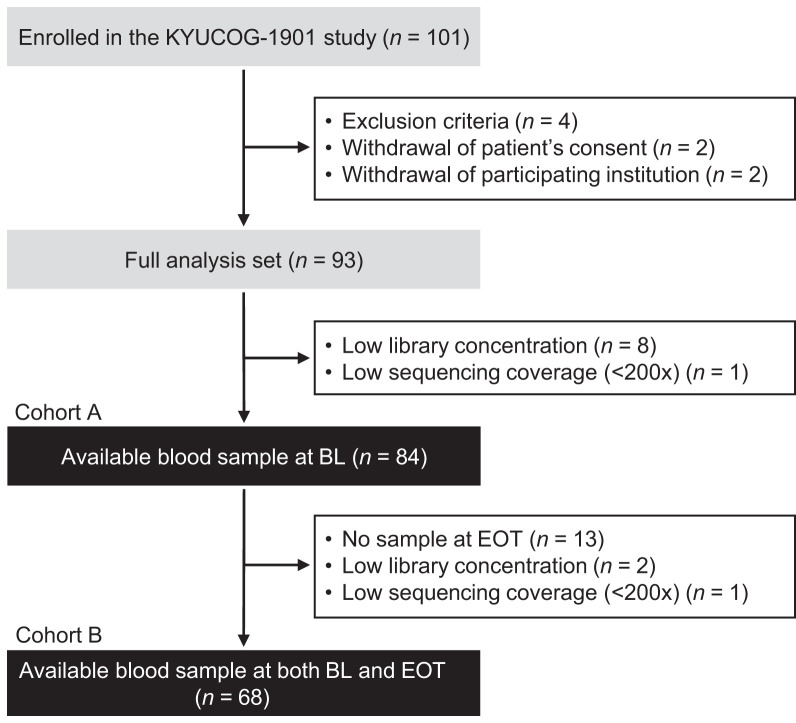
Consolidated Standards of Reporting Trials (CONSORT) diagram of patient and sample allocation. Patients were analyzed in 2 cohorts: cohort A (patients with available ctDNA samples at baseline, *n* = 84) and cohort B (patients with available ctDNA samples at both baseline and EOT, *n* = 68). BL = baseline.

Of 101 patients enrolled in the KYUCOG-1901 study, 93 patients were included in the full analysis set (Fig. 1). At baseline, ctDNA analysis was unsuccessful for 9 patients because of low library concentration (*n* = 8) or insufficient sequencing coverage (*n* = 1), leaving 84 patients (defined as cohort A) with evaluable baseline ctDNA data. Data on BSI changes from baseline to the end of treatment (EOT) were available for 52 patients in cohort A. At EOT, blood samples were not collected from 13 patients, and analysis failed for 3 patients (low library concentration in 2 patients and low coverage in 1 patient). Thus, paired baseline and EOT ctDNA data were available for 68 patients (defined as cohort B).

The baseline characteristics of cohorts A and B are shown in Table 1. The median age was 73 and 74 y in cohorts A and B, respectively. Asymptomatic or mildly symptomatic disease, determined as “worst pain in the past 24 h,” was 0 or 1–3 according to the Brief Pain Inventory and was present for 55 patients (65.5%) and 51 patients (75.0%) in cohorts A and B, respectively. The median baseline PSA level was 8.3 ng/mL in cohort A and 6.5 ng/mL in cohort B, and the median PSA doubling time was 2.3 and 2.1 mo, respectively. Extent of disease scores 1, 2, 3, and 4 were observed in 39.3%, 38.1%, 15.5%, and 7.1% of cohort A and in 38.2%, 36.8%, 17.6%, and 7.4% of cohort B, respectively. Prior treatment with androgen receptor signaling inhibitors and taxanes was administered in approximately 80% and 30% of patients, respectively.

**TABLE 1.** Patient Characteristics

Characteristic	Cohort A	Cohort B
No. of patients	84	68
Median age at baseline (y)	73 (68–77)	74 (68–77)
ECOG performance status at baseline		
0	62 (73.8)	53 (77.9)
1	20 (23.8)	14 (20.6)
2	1 (1.2)	1 (1.5)
Not available	1 (1.2)	0
Pain at baseline		
Asymptomatic or mild symptomatic	55 (65.5)	51 (75.0)
Symptomatic	26 (31.0)	16 (23.5)
Not available	3 (3.6)	1 (1.5)
Median PSA level at baseline (ng/mL)	8.3 (2.1–38.3)	6.5 (1.9–34.1)
Median PSA doubling time at baseline (mo)	2.3 (1.3–4.3)	2.1 (1.3–4.3)
Median hemoglobin level at baseline (g/dL)	12.8 (11.9–13.7)	12.7 (11.9–13.5)
Median ALP level at baseline (U/L)	90 (67–134)	89 (67–130)
Not available	1	1
Median LDH level at baseline (U/L)	199 (176–234)	197 (174–229)
Not available	3	2
ISUP grade group		
≤3	13 (15.5)	10 (14.7)
4	20 (23.8)	17 (25.0)
5	51 (60.7)	41 (60.3)
Prior local treatment		
Absence	51 (60.7)	42 (61.8)
Curative local treatment	33 (39.3)	26 (38.2)
T stage at diagnosis		
Tx	1 (1.2)	1 (1.5)
T1/2	32 (36.6)	25 (36.8)
T3	33 (39.3)	27 (39.7)
T4	18 (19.4)	15 (22.1)
N stage at diagnosis		
N0	44 (52.4)	39 (57.4)
N1	40 (47.6)	29 (42.6)
M stage at diagnosis		
M0	29 (34.5)	23 (33.8)
M1a	1 (1.2)	1 (1.5)
M1b	54 (64.3)	44 (64.7)
Lymph node metastasis at baseline		
Absence	81 (96.4)	66 (97.1)
Presence	3 (3.6)	2 (2.9)
EOD score at baseline		
1	33 (39.3)	26 (38.2)
2	32 (38.1)	25 (36.8)
3	13 (15.5)	12 (17.6)
4	6 (7.1)	5 (7.4)
Prior ARSI treatment		
Absence	15 (17.9)	14 (20.6)
Presence	69 (82.1)	54 (79.4)
Prior taxane treatment		
Absence	57 (67.9)	46 (67.6)
Presence	27 (32.1)	22 (32.4)
Concomitant treatment		
ADT monotherapy	41 (48.8)	33 (48.5)
ADT plus bicalutamide	5 (6.0)	5 (7.4)
ADT plus flutamide	5 (6.0)	5 (7.4)
ADT plus abiraterone	3 (3.6)	2 (2.9)
ADT plus enzalutamide	13 (15.5)	9 (13.2)
ADT plus others	17 (20.2)	14 (20.6)
Bone-modifying agent		
None	45 (53.6)	38 (55.9)
Denosumab	36 (42.9)	27 (39.7)
Zoledronate acid	3 (3.6)	3 (4.4)

ECOG = Eastern Cooperative Oncology Group; LDH = lactate dehydrogenase; ISUP = International Society of Urological Pathology; EOD = extent of disease; ARSI = androgen receptor signaling inhibitor; ADT = androgen deprivation therapy.

Categoric data are number followed by percentage in parentheses; continuous data are number followed by IQR in parentheses.

### Genomic Landscape in Baseline ctDNA

**FIGURE 2.**
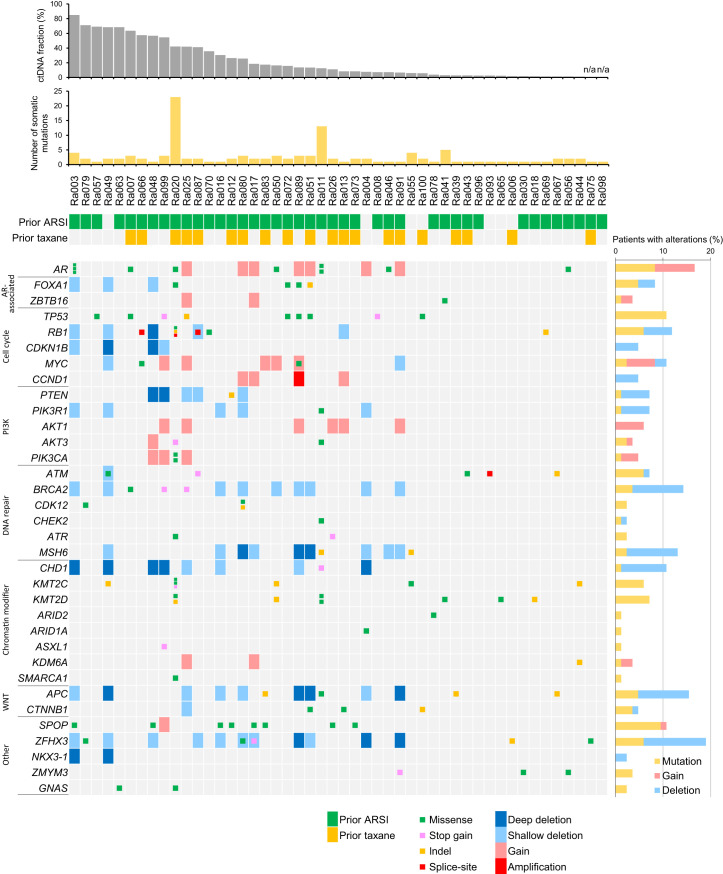
Integrative genomic landscape at baseline. Matrix summarizes genomic aberrations identified by cfDNA and paired leukocyte DNA sequencing in ctDNA detectable samples (*n* = 48). Deleterious somatic and germline variants, as well as copy number alterations, are color-coded by aberration type. Columns represent individual patient samples, sorted by ctDNA fraction (top graph). Rows indicate specific genes grouped by pathway, with cohort-wide alteration frequencies shown on right among 84 baseline samples. Number of somatic mutations per patient and prior treatments are shown in second graph and 2 rows below it, respectively. AR = androgen receptor; ARSI = androgen receptor signaling inhibitor; indel = insertion or deletion; n/a = not available; PI3K = phosphoinositide 3 kinase.

Among cohort A, the median cfDNA yield at baseline was 8.7 ng/mL. ctDNA was detectable in 48 patients (57.1%) but undetectable in 36 patients (42.9%). The median ctDNA fraction in 48 samples was 11.7% (range, 1.1%–84.9%). The baseline genomic landscape is shown in Figure 2, and individual gene alterations are listed in Supplemental Tables 2 and 3. Somatic *AR* mutations and *AR* copy number gain were each observed in 7 patients. *TP53*, *RB1*, and *PTEN* alterations were detected in 9, 9, and 11 patients, respectively. *BRCA2* mutations were observed in 3 patients (1 germline and 2 somatic), whereas no *BRCA1* mutation was detected. *SPOP* mutations were identified in 8 patients.

**FIGURE 3.**
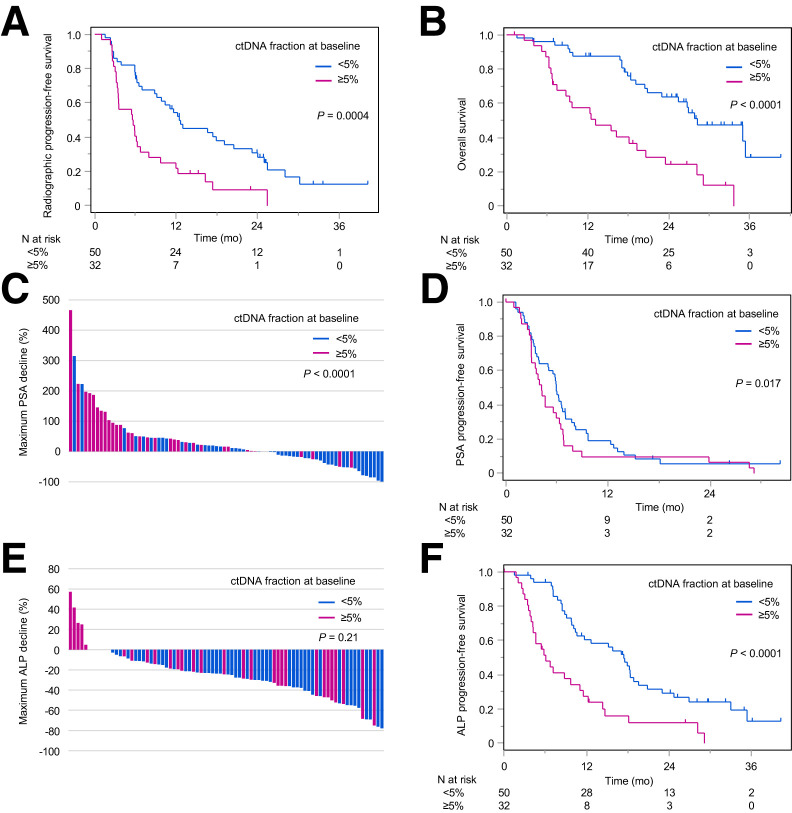
Prognostic impact of baseline ctDNA fraction. rPFS (A) and OS (B) stratified by ctDNA fraction (≥5% vs. <5%). Maximum PSA decline (C) and PSA-PFS (D) stratified by ctDNA fraction. Maximum ALP decline (E) and ALP-PFS (F) stratified by ctDNA fraction.

We then assessed the association of baseline ctDNA features with outcomes of ^223^Ra treatment. The ctDNA fraction was associated with shorter rPFS (HR, 3.27; 95% CI, 1.35–7.09; *P* = 0.0047) and OS (HR, 5.17; 95% CI, 1.84–12.80; *P* = 0.0008) when analyzed as continuous variables. Next, the analyses were performed by dichotomizing patients using a 5% threshold for the ctDNA fraction, determined based on PSA decline. An elevated ctDNA fraction was significantly associated with shorter rPFS (HR, 2.44; 95% CI, 1.47–4.08; *P* = 0.0006) and OS (HR, 3.30; 95% CI, 1.83–5.94; *P* < 0.0001) (Table 2; Figs. 3A and 3B). In multivariate analysis incorporating extent of disease, prior systemic therapies (androgen receptor signaling inhibitor and taxane), and baseline ALP levels, the elevated ctDNA fraction remained independently associated with shorter rPFS (HR, 2.50; 95% CI, 1.42–4.37; *P* = 0.0014) and OS (HR, 3.11; 95% CI, 1.63–5.91; *P* = 0.0006). A higher ctDNA fraction was also associated with less PSA decline and shorter PSA-PFS, shorter ALP-PFS, more frequent early discontinuation of ^223^Ra before completing 6 cycles (12.0% in the low ctDNA fraction and 43.8% in the high ctDNA fraction, *P* = 0.0016; data not shown), and less BSI decline, but not with ALP decline (Figs. 3C–3F; Supplemental Fig. 2A). However, the ctDNA fraction was not significantly correlated with BSI (*r* = 0.08, *P* = 0.54). Meanwhile, PSA of at least 10 versus less than 10 for rPFS (HR, 1.42; 95% CI, 0.88–2.28; *P* = 0.15) and for OS (HR, 1.45; 95% CI, 0.83–2.53; *P* = 0.19) and ALP of at least 90 versus less than 90 for rPFS (HR, 1.37; 95% CI, 0.85–2.22; *P* = 0.20) and for OS (HR, 1.71; 95% CI, 0.97–3.02; *P* = 0.062) at baseline were not associated with rPFS or OS (data not shown).

**TABLE 2.** Association Between ctDNA Profile at Baseline and rPFS

			Univariate analysis			Adjusted with ctDNA fraction		
Profile	*n*	Median duration (mo)	HR	95% CI	*P*	HR	95% CI	*P*
Total cohort	84	8.8						
ctDNA fraction ≥ 5%	32	5.6	2.44	1.47–4.08	0.0006*			
Altered gene								
*AR*	14	5.7	1.75	0.95–3.21	0.074	1.44	0.75–2.78	0.27
*TP53*	9	3.4	2.65	1.30–5.42	0.0077*	2.46	1.19–5.09	0.016*
*RB1*	9	3.3	2.94	1.42–6.10	0.0037*	2.04	0.73–5.69	0.17
*PTEN*	11	3.2	5.79	2.78–12.00	<0.0001*	4.08	1.62–10.20	0.0028*
*BRCA2*	12	4.5	1.71	0.89–3.29	0.11	1.28	0.62–2.66	0.51
*MSH6*	9	6.2	1.06	0.48–2.33	0.88	1.07	0.48–2.34	0.87
*CHD1*	9	3.5	2.10	1.03–4.25	0.04*	1.47	0.64–3.37	0.37
*APC*	13	6.2	1.49	0.79–2.79	0.21	1.22	0.62–2.38	0.56
*SPOP*	9	5.5	2.45	1.19–5.08	0.015*	1.76	0.75–4.16	0.20
*ZFHX3*	10	5.6	2.07	1.01–4.26	0.047*	1.17	0.43–3.20	0.76
Altered pathway								
AR-associated	9	3.1	2.70	1.32–5.55	0.0067*	1.94	0.82–4.63	0.13
Cell cycle	22	3.5	3.47	2.00–6.03	<0.0001*	3.50	1.79–6.85	0.0003*
PI3K	16	3.5	2.26	1.26–4.06	0.0063*	1.86	0.96–3.61	0.066
DNA repair	23	5.7	1.53	0.89–2.62	0.12	1.21	0.65–2.25	0.54
Chromatin modifier	18	6.0	1.73	0.99–3.02	0.055	1.46	0.80–2.68	0.22
WNT	15	6.2	1.50	0.83–2.72	0.18	1.28	0.69–2.40	0.44

* Statistically significant.

AR = androgen receptor; PI3K = phosphoinositide 3 kinase.

**FIGURE 4.**
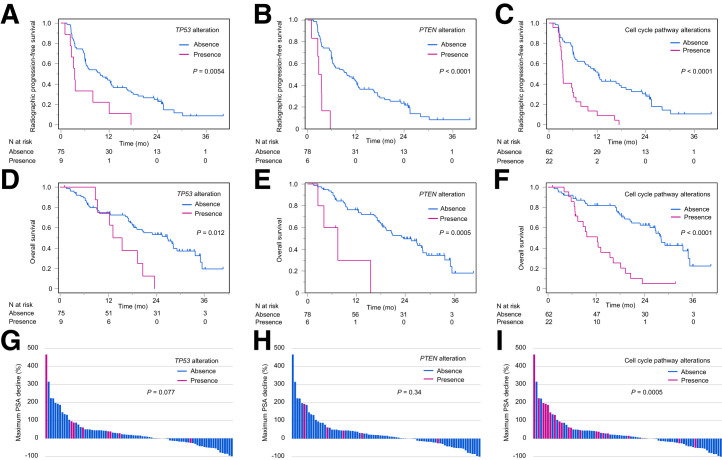
Prognostic impact of baseline ctDNA gene signatures. rPFS stratified by *TP53* alteration (A), *PTEN* alteration (B), and cell cycle pathway alterations (C). OS stratified by *TP53* alteration (D), *PTEN* alteration (E), and cell cycle pathway alterations (F). Maximum PSA decline stratified by *TP53* alteration (G), *PTEN* alteration (H), and cell cycle pathway alterations (I).

*TP53* alteration (HR, 2.46; 95% CI, 1.19–5.09; *P* = 0.016) and *PTEN* alteration (HR, 4.41; 95% CI, 1.68–11.60; *P* = 0.0027) were associated with worse rPFS, independent of ctDNA fraction (Table 2; Figs. 4A and 4B). When the oncogenic pathway was grouped as indicated in Figure 2, alterations in the cell cycle pathway (HR, 3.50; 95% CI, 1.79–6.85; *P* = 0.0003) were associated with inferior rPFS, independent of ctDNA fraction (Table 2; Fig. 4C). *TP53* alteration, *PTEN* alteration, and the cell cycle pathway alterations were also associated with poorer OS (Figs. 4D–4F). Similarly, cell cycle pathway alterations were associated with poorer PSA response, although statistical significance was not reached in *TP53* alteration and *PTEN* alteration (Figs. 4G–4I). In addition, *TP53* alteration (24.0% in the absence of alterations and 44.4% in the presence of alterations, *P* = 0.021), *PTEN* alteration (23.1% in the absence of alterations and 66.7% in the presence of alterations, *P* = 0.038), and cell cycle pathway alterations (17.7% in the absence of alterations and 43.8% in the presence of alterations, *P* = 0.0051) were associated with more frequent early discontinuation of ^223^Ra (data not shown). Cell cycle pathway alterations were associated with less BSI decline, whereas *TP53* alteration and *PTEN* alteration were not (Supplemental Figs. 2B–2D). *TP53* alteration, *PTEN* alteration, and cell cycle pathway alterations were associated with shorter time to visceral metastasis (data not shown).

### Change of ctDNA Profiles After ^223^Ra Treatment

**FIGURE 5.**
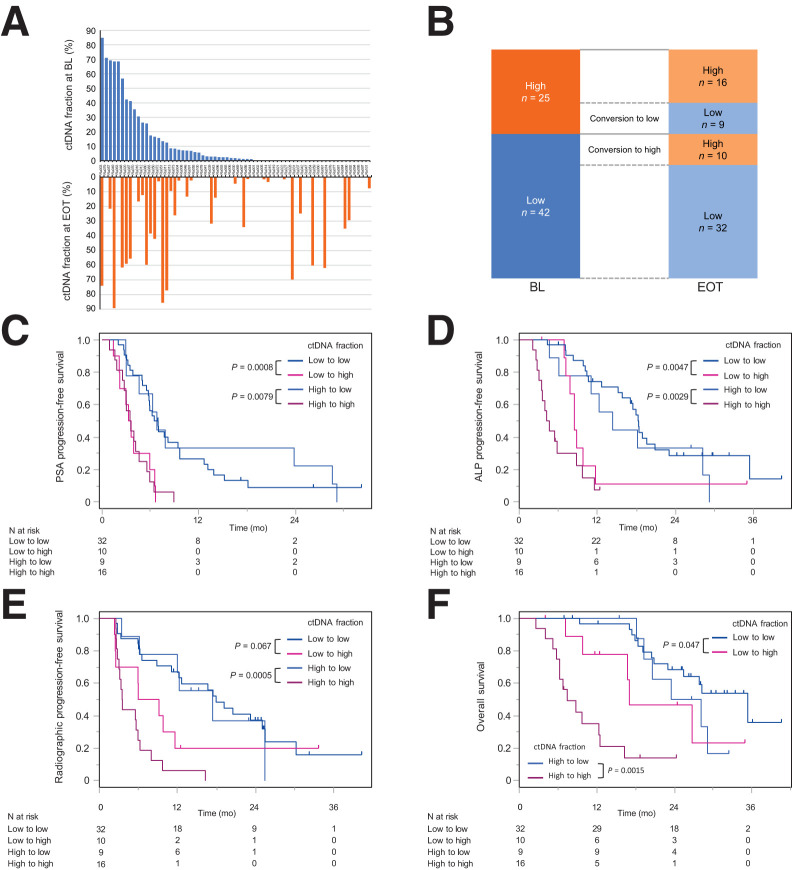
Changes in ctDNA profile from baseline to EOT and prognostic impact. (A) Change in ctDNA fraction in paired baseline and EOT samples from same patients. (B) Proportions of patients with low (<5%) and high (≥5%) ctDNA fraction at baseline and EOT. PSA-PFS (C), ALP-PFS (D), rPFS (E), and OS (F) according to changes in ctDNA fraction between baseline and EOT. BL = baseline.

Next, we analyzed paired baseline and EOT samples in cohort B (*n* = 68). The cfDNA concentration significantly increased at EOT (median, 16 ng/mL; IQR, 8.2–80.0) compared with baseline (median, 9.6 ng/mL; IQR, 5.1–18.0; *P* = 0.0027). However, the ctDNA fraction remained largely unchanged between baseline (median, 1.7%; IQR, 0–11) and EOT (median, 1.4%; IQR, 0–29; *P* = 0.83; Fig. 5A). Among patients with a low ctDNA fraction (<5%) at baseline, 76.2% remained low and 23.8% converted to high at EOT. Among those with a high ctDNA fraction (≥5%) at baseline, 64.0% remained high and 36.0% converted to low (Fig. 5B). Although the number of somatic mutations did not significantly change (*P* = 0.57), the number of copy number variations significantly increased after ^223^Ra (*P* < 0.0001).

With regard to changes in gene signatures between baseline and EOT, the VAF of each mutation, normalized to the ctDNA fraction, was compared among patients with ctDNA detected at both time points. Although mutation VAFs were positively correlated between baseline and EOT (*r* = 0.5; Supplemental Fig. 3), several gene mutations exhibited dynamic changes during ^223^Ra treatment (Supplemental Table 4). Among *AR*, *TP53*, *RB1*, and *PTEN* alterations, *PTEN* alteration emerged in 5 cases, increasing from 3 cases at baseline to 11 cases at EOT (*P* = 0.025), whereas no significant changes were observed in *AR* (*P* = 1.00), *TP53* (*P* = 0.058), or *RB1* (*P* = 0.41) alteration. In addition, the emergence of *PTEN* alteration was associated with shorter time to visceral metastasis (data not shown), as well as shorter rPFS and OS (Supplemental Figs. 4A and 4B).

Finally, we analyzed the association of ctDNA dynamics with outcomes. Conversion from a high to a low ctDNA fraction at EOT was associated with improved PSA-PFS, ALP-PFS, rPFS, and OS (Figs. 5C–5F). Conversely, conversion from a low to a high ctDNA fraction was associated with worse PSA-PFS, ALP-PFS, and OS, with a marginal effect on rPFS. By comparison, maximal PSA decline (≥50% vs. <50%) was associated with rPFS but not OS (Supplemental Figs. 5A and 5B), and maximal ALP decline (≥50% vs. <50%) was associated with neither rPFS nor OS (Supplemental Figs. 5C and 5D).

## DISCUSSION

In this prospective, observational, multicenter study (KYUCOG-1901), we investigated the genomic landscape of ctDNA and its clinical utility as a predictive and monitoring biomarker in patients with bone mCRPC treated with ^223^Ra. To our knowledge, this is the first study to prospectively evaluate ctDNA dynamics and their association with clinical outcomes in the context of ^223^Ra therapy, after 1 small study that enrolled 28 patients (23).

Our findings yielded several important insights. First, a higher baseline ctDNA fraction (≥5%) was significantly associated with shorter rPFS and OS. This is consistent with prior studies, which found that a higher ctDNA burden correlated with worse prognosis, irrespective of the treatment (9–11,22). These results support the value of the ctDNA fraction as a robust prognostic marker even in the setting of ^223^Ra therapy and underscore the utility of liquid biopsy for identifying high-risk patients before initiating ^223^Ra therapy. A recent prospective, observational study, albeit with a small sample size, also showed that fewer circulating tumor cells were associated with better OS during ^223^Ra treatment (23).

In addition, deleterious alterations in the key tumor suppressor genes *TP53* or *PTEN* and cell cycle pathway alterations were significantly associated with poor outcomes, independent of ctDNA fraction. These alterations also correlated with higher rates of early discontinuation of ^223^Ra, suggesting that molecular profiling could help identify patients unlikely to complete the planned treatment course. These findings are consistent with the established role of these genes in driving aggressive disease biology and resistance to androgen receptor–targeted therapies (24,25). Alterations in *TP53* or *PTEN* and cell cycle pathway alterations are known to be enriched in advanced stages of prostate cancer and associated with poor prognosis (24,26–28). Similarly, a retrospective study of 124 patients with mCRPC from the Mayo Clinic and Tulane Cancer Center reported that *RB1* deletions were associated with worse PFS and OS in ^223^Ra treatment (29). By contrast, our study did not confirm the previously suggested association between DNA damage repair defects and favorable outcomes with ^223^Ra, as reported in earlier retrospective studies with small cohorts (30–32).

From a biologic perspective, we observed an increase of clone with *PTEN* alteration at the EOT, which was strongly associated with poor prognosis in ^223^Ra treatment. *PTEN* loss has been linked to ferroptosis suppression, mitochondrial redox buffering, and hypoxia-driven metabolic rewiring, each of which can protect tumor cells from radiation-induced oxidative damage. These attributes may be particularly relevant in the bone metastatic niche, in which hypoxia and osteoblastic activity create a permissive microenvironment for treatment-resistant clones (33). *PTEN* alteration was also associated with the emergence of visceral metastases, whereas no clear association was observed with the progression of bone metastases. Consistently, we have recently shown that *PTEN* alteration detected by ctDNA analysis was associated with a higher frequency of lung metastases in the SCRUM-Japan MONSTAR SCREEN project (34). Taken together, these findings suggest that *PTEN*-deficient tumors may evade the therapeutic effects of ^223^Ra, ultimately leading to treatment failure, and warrant further investigation.

Dynamic changes in ctDNA during treatment appeared to reflect treatment response and disease trajectory. Specifically, patients whose ctDNA fraction decreased from high to low during treatment had longer rPFS and OS, whereas those with an increasing ctDNA fraction had worse outcomes. In addition, emergent *PTEN* alteration was associated with shorter rPFS and OS. Recently, a decrease in BSI was also shown to correlate with better OS in ^223^Ra treatment in a small prospective study (35). The combination of ctDNA and BSI monitoring may offer a more comprehensive assessment of treatment response, although further validation is needed.

These findings have several clinical implications. The detection of a high baseline ctDNA fraction, deleterious *TP53* or *PTEN* alterations, or cell cycle pathway alterations could help identify patients at high risk of poor outcomes who may benefit from alternative or intensified treatment strategies. Serial ctDNA monitoring could also enable early detection of emerging resistance, allowing timely adjustments in therapy before overt progression. However, the present findings alone cannot determine how ctDNA profiling should be incorporated into patient selection, sequencing of systemic therapies, or on-treatment monitoring for ^223^Ra in routine clinical practice, because this study did not involve ctDNA-guided clinical decision-making. Thus, future ctDNA-guided interventional trials will be needed to determine whether such strategies can optimize the use of ^223^Ra and improve outcomes.

Our study has some limitations. The sample size was moderate, and analyses of individual gene alterations were limited by small event numbers, especially in subgroup analysis. Although the ctDNA assay demonstrated high sensitivity and specificity, it may miss low-frequency subclones or alterations outside the targeted gene panel. Genomic subgroup comparisons raise the possibility of a type 1 error due to multiple testing. Given the hypothesis-generating nature and limited sample size, we opted not to apply formal multiplicity correction. Therefore, these exploratory analyses should be interpreted cautiously. The absence of an independent validation cohort represents an important limitation and restricts the generalizability of the proposed ctDNA thresholds and composite genomic features. External validation using larger, multiinstitutional cohorts will be required to determine whether these molecular profiles are reproducible and clinically actionable in ^223^Ra-treated mCRPC. Finally, as an observational study without a control arm, causality cannot be definitively established. Nevertheless, a key strength of our study is its prospective design with protocolized frequent monitoring.

## CONCLUSION

This prospective, observational study suggests the clinical utility of ctDNA profiling as both a prognostic and a monitoring tool for patients with bone mCRPC treated with ^223^Ra. The baseline ctDNA fraction and key genomic alterations are associated with treatment outcomes, and dynamic changes in ctDNA during ^223^Ra therapy reflect disease trajectory. Further large-scale studies are warranted to validate these findings and to explore ctDNA-guided therapeutic strategies for improving outcomes in ^223^Ra treatment.

## DISCLOSURE

This work was conducted with financial support to the Clinical Research Support Center Kyushu (Fukuoka, Japan) from Bayer Yakuhin. The funder had no role in the study design and was not involved in its conduct, data analysis, or interpretation, or in the decision to submit the results for publication. Masaki Shiota received honoraria from Janssen Pharmaceutical, AstraZeneca, Astellas Pharma, Sanofi, and Bayer and research funding support from Astellas Pharma. Tomomi Kamba received honoraria from MSD and Takeda Pharmaceuticals. Tsukasa Igawa received honoraria from Bayer. Naoya Masumori received honoraria from Bayer. Shusuke Akamatsu received honoraria from Bayer, Janssen Pharmaceutical, Astellas Pharma, and Takeda Pharmaceuticals and research funding support from Tosoh Corp. Masatoshi Eto received honoraria from Takeda Pharmaceutical, Janssen Pharmaceutical, AstraZeneca, and Astellas Pharma and research funding support from Astellas Pharma, Sanofi, and Takeda Pharmaceutical. No other potential conflict of interest relevant to this article was reported.

## Notes

KEY POINTS **QUESTION:** Can ctDNA profiling serve as a reliable biomarker to predict and monitor treatment outcomes in patients with bone mCRPC receiving ^223^Ra therapy? **PERTINENT FINDINGS:** In this prospective, multicenter, observational study (KYUCOG-1901), ctDNA was successfully analyzed in 84 baseline and 74 EOT plasma samples from 93 patients treated with ^223^Ra. A baseline ctDNA fraction of at least 5% and deleterious alterations in *TP53* or *PTEN*, as well as cell cycle pathway alterations, were significantly associated with shorter rPFS and OS. Moreover, dynamic changes in the ctDNA fraction and *PTEN* alteration during ^223^Ra treatment correlated more strongly with clinical outcomes. **IMPLICATIONS FOR PATIENT CARE:** ctDNA profiling provides a minimally invasive means to identify patients at high risk of poor outcomes before initiating ^223^Ra and to monitor molecular response during therapy. The incorporation of ctDNA fraction and genomic alterations into clinical practice may refine patient selection, enable early detection of treatment resistance, and ultimately optimize personalized management strategies for patients with bone mCRPC undergoing ^223^Ra therapy.
